# Reliability and construct validity of the Spanish version of the 6-item CTS symptoms scale for outcomes assessment in carpal tunnel syndrome

**DOI:** 10.1186/s12891-016-0963-5

**Published:** 2016-03-03

**Authors:** Roberto S. Rosales, Yolanda Martin-Hidalgo, Luis Reboso-Morales, Isam Atroshi

**Affiliations:** Unit for Hand & Microsurgery, GECOT, La Laguna, Tenerife Spain; Department of Orthopedics, University Hospital of La Candelaria, Santa Cruz de Tenerife, Spain; Departments of Clinical Sciences, Lund University, Lund, Sweden; Orthopedics Hässleholm-Kristianstad, Hässleholm Hospital, Hässleholm, Sweden

**Keywords:** Carpal tunnel syndrome, CTS-6 symptoms scale, Patient-reported outcomes, Carpal tunnel release, QuickDASH

## Abstract

**Background:**

The purpose of this study was to assess the reliability and construct validity of the Spanish version of the 6-item carpal tunnel syndrome (CTS) symptoms scale (CTS-6).

**Methods:**

In this cross-sectional study 40 patients diagnosed with CTS based on clinical and neurophysiologic criteria, completed the standard Spanish versions of the CTS-6 and the disabilities of the arm, shoulder and hand (QuickDASH) scales on two occasions with a 1-week interval. Internal-consistency reliability was assessed with the Cronbach alpha coefficient and test-retest reliability with the intraclass correlation coefficient, two way random effect model and absolute agreement definition (ICC_2,1_). Cross-sectional precision was analyzed with the Standard Error of the Measurement (SEM). Longitudinal precision for test-retest reliability coefficient was assessed with the Standard Error of the Measurement difference (SEMdiff) and the Minimal Detectable Change at 95 % confidence level (MDC_95_). For assessing construct validity it was hypothesized that the CTS-6 would have a strong positive correlation with the QuickDASH, analyzed with the Pearson correlation coefficient (r).

**Results:**

The standard Spanish version of the CTS-6 presented a Cronbach alpha of 0.81 with a SEM of 0.3. Test-retest reliability showed an ICC of 0.85 with a SRMdiff of 0.36 and a MDC_95_ of 0.7. The correlation between CTS-6 and the QuickDASH was concordant with the a priori formulated construct hypothesis (r 0.69)

**Conclusions:**

The standard Spanish version of the 6-item CTS symptoms scale showed good internal consistency, test-retest reliability and construct validity for outcomes assessment in CTS. The CTS-6 will be useful to clinicians and researchers in Spanish speaking parts of the world. The use of standardized outcome measures across countries also will facilitate comparison of research results in carpal tunnel syndrome.

**Electronic supplementary material:**

The online version of this article (doi:10.1186/s12891-016-0963-5) contains supplementary material, which is available to authorized users.

## Background

The use of disease-specific measures of patient-reported outcomes (PRO) has grown in clinical research. Carpal tunnel syndrome (CTS) is one of the most frequent conditions managed at hand surgery services. The CTS questionnaire developed by Levine et al. [1] has been among the most widely used PRO measures during the last two decades. The CTS questionnaire consists of two scales: symptoms severity (SS) (11 items) and functional status (FS). Atroshi et al. [2], using factor analysis and Items Response Theory methodology, developed a short version of the CTS SS-scale consisting of 6 items with the purpose of reducing respondent burden while maintaining the scale's psychometric properties. They have demonstrated that the new brief version possessed a good level of reliability, validity and responsiveness [2–4]. Because a Spanish version of the 11-item symptom severity scale was already available [5], a Spanish version of the shorter version (CTS-6) was introduced [6]. No new evidence has been reported about the reliability and validity of the CTS-6 since the first description done by Atroshi et al. [2] in 2009.

The purpose of this study was to assess the reliability and construct validity of the Spanish version of the 6-items CTS symptoms scale for outcomes assessment in CTS.

## Methods

### Study population

All procedures performed in this study involving human participants were in accordance with the ethical standards of the institutional national research committee of the University Hospital of La Candelaria, School of Medicine, University of La Laguna and with the 1964 Helsinki declaration and its later amendments or comparable ethical standards. The ethic committe reviewed and approved this study. Written informed consent was obtained from all individual participants included in the study.

#### Eligibility criteria

The inclusion criteria were: (1) numbness or tingling with or without pain in at least 2 of the 4 radial digits [7, 8], (2) increased symptoms with carpal tunnel provocative tests (positive Phalen and/or reverse Phalen test) [8], (3) symptoms duration of more than two months [7], (4) failure of conservative treatment [7], and nerve conduction test showing median neuropathy at the wrist (distal motor latency > 4.5 milliseconds, wrist-digit sensory latency > 3.5 milliseconds, or sensory conduction velocity at the carpal tunnel segment < 40 m/s) [9, 10]. The exclusion criteria were clinical or electrophysiological signs of proximal nerve compression, diabetes or other metabolic disease, and rheumatoid arthritis or other general inflammatory diseases [7, 8, 11].

#### Recruitment and enrollment

The study was conducted at a single center, orthopaedic department, University Hospital of La Candelaria. Patients were recruited among those referred by primary care doctors because of symptoms of CTS. Eligible patients were enrolled by the examining orthopaedic-hand surgeons (YMH, LRM) after thorough clinical examination and nerve conduction study. Patients who met the eligibility criteria were scheduled for surgery and invited to participate in the study. Each patient was given verbal and written information about the study and informed consent was obtained.

The study population consisted of 40 consecutive patients with the diagnosis the CTS and waiting for carpal tunnel release in the National Health System, Tenerife, Spain (Table 1)

**Table 1:** Patients and demographic characteristics

Study Population (*N* = 40)	
Age (mean, SD)	54 (11)
Gender (male/female)	14/26
Affected Hand (right/left)	27/13
Baseline CTS-6 score^a^ (mean, SD)	3.7 (0.7)
Baseline QucikDASH score^b^ (mean, SD)	63.9 (19.5)

^a^CTS-6 (symptoms severity) range of scores from 1 (no symptoms) to 5 (most severe symptoms)

^b^QuickDASH (upper extremity disability) range of scores from 0 (no disability) to 100 (most severe disability)

.

### Clinical design

A cross-sectional study which adhered to STROBE guidelines (Additional file 1).

### Outcomes measures

The standard Spanish versions of the CTS-6 [6] and the 11-item disabilities of the arm, shoulder and hand (QuickDASH) questionnaire (www.dash.iwh.on.ca) were completed by the patients at the outpatient clinic.

The CTS-6 measures symptoms severity related to CTS. It consists of 6 items. Five of the 6 items in the CTS-6 have similar item text as the corresponding items in the original 11-item symptom severity scale and the remaining item (the result of merger of 2 symptom severity scale items) has text from the 2 items. The CTS-6 has, however, a completely different and improved layout [2]. The scoring is similar to that for the 11-item symptom severity scale; for each patient the item responses are scored from 1 (best) to 5 (worst) and then averaged for the 6 items to yield a CTS-6 score (only 1 missing item response is allowed).

The QuickDASH is the shorter version of the DASH PRO measure [12] developed for measuring “upper extremities disability”. It consists of 11 items and it is scored from 0 (best) to 100 (worst). At least 10 of the 11 items must be completed for a score to be calculated. Each item is scored 1 to 5 and the assigned values for all completed items are summed and averaged, producing a score of 1 to 5. This value is then transformed to a score of 0 to 100 by subtracting one and multiplying by 25. This transformation is done to make the score easier to compare to other measures scaled on a 0–100 scale.

No missing items from the two PRO instruments were observed in this study.

For assessing test-retest reliability a second self administration of the CTS-6 was done at the clinic 1 week after the first administration.

### Data analysis

Internal-consistency reliability was assessed with the Cronbach alpha coefficient (alpha > 0.7 indicates good internal consistency). Test-retest reliability was assessed with the intraclass correlation coefficient, two way random effect model and absolute agreement definition (ICC_2,1_) and by comparing the mean CTS-6 scores for the two consecutive administrations with the paired *t*-test. For the test-retest reliability mean difference analysis, a sample of 19 individiuals will be needed to detect an important clinical difference of 0.9 in the CTS-6 scores, assuming a SD of 0.7 [2], two-sided test, power of 80 %, and a level of significance of 0.05. Cross- sectional precision was analyzed based on the Standard Error of the Measurement (SEM). Longitudinal precision for test-retest reliability coefficient was assessed with the Standard Error of the Measurement difference (SEMdiff) and the Minimal Detectable Change at 95 % confidence level (MDC_95_). For assessing construct validity it was hypothesized that the CTS-6 scores would have a moderate to strong positive correlation with the QuickDASH. The construct validity hypothesis was analyzed with the Pearson correlation coefficient (r), using a level 0.01 for statistical significance (values between 0. 8 and 1.0 indicating a very strong relationship, between 0.6 and 0.8 a strong relationship, between 0.4 and 0.6 a moderate relationship, between 0.2 and 0.4 a weak relationship, and less than 0.2 very weak or no relationship) [13]. All parametric tests used in the analysis was based on the assumption of the data was normally distributed after exploration.

## Results

### Reliability

The Cronbach alpha coefficient was 0.81 with SEM of 0.3, and the ICC was 0.85 (*p* < 0.001) with SRMdiff of 0.36 and a MDC_95_ of 0.7. The mean difference in the CTS-6 scores for the two administration times was 0.09 (95 % confidence interval −0.07 to 0.26, *p* = 0.27) (Table 2).

**Table 2:** Results of the CTS-6 Reliability Analysis (*N* = 40)

Internal consistency. Calculated SEM and cross-sectional precision estimates for CTS-6 scores using Cronbach alpha coefficient and SD observed during field testing			
Cronbach alpha coefficient	SD of CTS-6 scores	SEM	Range of cross-sectional precision of CTS-6 scores 95 % confidence level
0.81	0.68	0.3	Observed score +/−0.59
Test-retest reproducibility. Calculated longitudinal SEM of differences (*SEM diff*) based on test-retest reliability coefficient (ICC_2,1_) and the SD of baseline scores			
Mean difference score (95 % CI)^a^	ICC_(2,1)_ (95 % CI)^a^	SEMdiff	MDC (95)
0.09 (−0.07–0.26)	0.85 (0.71–0.92)	0.36	0.70

*ICC*_*(2,1)*_ Intraclass correlation coefficient: two-way random effect model (absolute agreement definition), *MDC (95)* minimal detectable change 95 % CI

^a^Mean difference between scores recorded in 2 successive administration times 1 week apart. CI = confidence interval

### Construct validity

There was a strong positive correlation between the scores for the CTS-6 scale and the QuickDASH (*r* = 0.69, *p* < 0.001),

## Discussion

The results have demonstrated that the CTS-6 PRO measure has good internal consistency and test-retest reliability with a mean difference of the 1-week test-retest scores not statistically different from zero and lower than the MDC_95_. A high level of intraclass correlation that meets the minimal standards for reliability analysis was observed. Besides, the correlations were concordant with the construct hypothesis formulated a priori, supporting construct validity.

One of the measurement properties of PRO instruments included by the Medical Outcomes Trust in the instruments review criteria is the “respondent burden" defined as the time, energy, and other demands placed on those to whom the instrument is administered [14]. The CTS-6 was developed to improve patient acceptance, to increase response rate and consequently the efficiency of the scale while maintaining good psychometric properties [3].

In this study, the Spanish CTS-6 presented a Cronbach alpha coefficient of 0.81 and an ICC of 0.85. Similar results have been reported with the original CTS-6 (Cronbach alpha = 0.86, ICC = 0.95) [2]. The mean difference of scores in two administration times in the original CTS-6 was 0.03 (95 % CI −0.07 to 0.12) [2]. In the present study the mean difference in the test-retest scores was 0.09 (95 % CI −0.07 to 0.26), lower than the MDC_95_. (0.7). The test-retest reliability results are similar to the reliability of the longer Spanish version (11-items symptom severity scale), with the same “washout time” of 1 week (mean difference in scores = 0.18, 95 % CI −0.16 to 0.53, *p* = 0.248) [5]. Consequently, the Spanish CTS-6 presents a level of reliability similar to the original CTS-6 and the Spanish CTS-11.

There are different aspects of validity of a health outcome measure: content validity, criterion-validity, and construct validity [14–16]. Common methods to assess construct-related validity include examination of the logical relationship that should exist between that measure and other measures and/or patterns of scores across groups of individuals. Testing for construct validity involves assessing both theory and method simultaneously. Therefore, it should include the hypothesis that can demonstrate the proposed construct (theory) [15, 16]. Many factors should be considered when choosing hypotheses. The most important factor is the specific dimension of health or concept that is intended to be measured by using a patient-completed questionnaire and the way or direction of scoring of every instrument studied. Atroshi et al. [2] demonstrated a convergent validity by a strong correlation between the original CTS-6 scores and the QuickDASH scores (*r* = 0.7). In this study we have observered a very similar correlation coefficient (*r* = 0.69) between the Spanish CTS-6 and QuickDASH. The results of this study showed that the prespecified construct hypothesis was established.

We did not perform a priori sample size calculation for the correlation analysis and it may be a limitation of this study. However, post hoc analysis has shown that based on the proposed null hypothesis (Ho = the correlations is equal zero), with a level of significance of 0.01, two-tailed test and the observed correlation coefficient of 0.69, a sample size of 20 patients would have a power of 80 % to yield a statistically significant result [17].

Measurement Error Statistics (MES) can help clinicians and researchers decide on a best practice whether the observed scores or change in a patient´s performance is true. But, MES does not provide information about which minimal change in CTS-6 scores is related to an important clinical improvement for the patients, called as “Minimal Clinically Importance Difference (MICD)”. Consequently, further studies regarding responsiveness and MCID are recommended to complete the analysis of the measurements properties of the Spanish CTS-6.

## Conclusions

This study demonstrates that the Spanish CTS-6 has good reliability and construct validity for outcomes assessment in CTS. The CTS-6 will be useful to clinicians and researchers in Spanish speaking parts of the world. The use of standardized outcome measures across countries also will facilitate comparison of research results in carpal tunnel syndrome.

## Additional file

Additional file 1: STROBE Statement—Checklist of items that should be included in reports of cross-sectional studies. (DOCX 30 kb)
